# Developing an Educational Program on Aging for High School Students

**DOI:** 10.1093/geroni/igab046.2792

**Published:** 2021-12-17

**Authors:** Tracy Davis, Amanda Sokan

**Affiliations:** 1 Rutgers University, New Brunswick, New Jersey, United States; 2 The University of Arizona, University of Arizona, Arizona, United States

## Abstract

College students in disciplines that might provide services or work with older adults, such as medicine or social work, are usually the target of most educational programs on aging. High schools provide an untapped opportunity to engage students earlier. This project is the next step following a pilot study conducted in New Jersey and Kentucky to better understand high school students’ attitudes and knowledge regarding aging. That study also reviewed current high school curriculum for aging-specific content and perceived barriers among teachers to incorporating aging education into the curriculum (Davis & Sokan, 2019). Study findings indicate inter alia, a need to educate high school students about aging, increase interactions among older and younger adults, incorporate education about careers on aging, and educate teachers on how to infuse more aging content into their courses. To that end, this project’s goal was to develop both a training module and educational program on aging for high school students. Also, we propose a plan to develop, implement, and evaluate both the training module and the educational programs. We hypothesize that the training module will increase high school teachers’ confidence in their ability to teach their students about aging. The educational program’s delivery will increase students’ knowledge of aging-related issues and awareness about careers in aging. Upon completing the project, we will use feedback from students and teachers to revise the educational program, for implementation among a larger sample of high schools.

